# Environmentally evaluated HPLC-ELSD method to monitor enzymatic synthesis of a non-ionic surfactant

**DOI:** 10.1186/1752-153X-8-33

**Published:** 2014-05-20

**Authors:** Yasser Gaber, Cecilia Orellana Åkerman, Rajni Hatti-Kaul

**Affiliations:** 1 Department of Microbiology, Faculty of Pharmacy, Beni-Suef University, Salah Salem street, 62511 Beni-Suef, Egypt; 2 Department of Biotechnology, Center for Chemistry and Chemical Engineering, Lund University, P.O. Box 124, SE-221 00 Lund, Sweden

**Keywords:** Green analytical chemistry, Chromatographic separation, Evaporative light scattering detector, Non-ionic surfactant, Mass spectrometry, HPLC-EAT

## Abstract

**Background:**

N-Lauroyl-N-methylglucamide is a biodegradable surfactant derived from renewable resources. In an earlier study, we presented an enzymatic solvent-free method for synthesis of this compound. In the present report, the HPLC method developed to follow the reaction between lauric acid/methyl laurate and N-methyl glucamine (MEG) and its environmental assessment are described.

**Results:**

Use of ultraviolet (UV) absorption or refractive index (RI) detectors did not allow the detection of N-methyl glucamine (MEG). With Evaporative light scattering detector ELSD, it was possible to apply a gradient elution, and detect MEG with a limit of detection, LOD = 0.12 μg. A good separation of the peaks: MEG, lauric acid, product (amide) and by-product (amide-ester) was achieved with the gradient program with a run time of 40 min. The setting of ELSD detector was optimized using methyl laurate as the analyte. LC-MS/MS was used to confirm the amide and amide-ester peaks. We evaluated the greenness of the developed method using the freely available software HPLC-Environmental Assessment Tool (HPLC-EAT) and the method got a scoring of 73 HPLC-EAT units, implying that the analytical procedure was more environmentally benign compared to some other methods reported in literature whose HPLC-EAT values scored up to 182.

**Conclusion:**

Use of ELSD detector allowed the detection and quantification of the substrates and the reaction products of enzymatic synthesis of the surfactant, N-lauroyl-N-methylglucamide. The developed HPLC method has acceptable environmental profile based on HPLC-EAT evaluation.

## Background

**Scheme 1:**
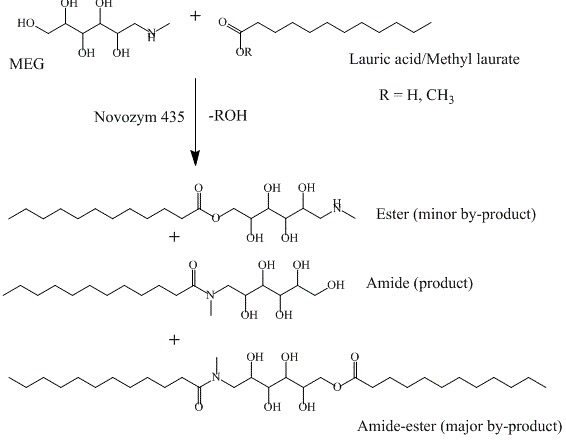
Lipase-mediated synthesis of N-lauroyl-N-methyl glucamide.

N-Acyl-N-methyl glucamides, also referred to as fatty acid glucamides or alkyl glucamides (AGs), are non-ionic surfactants derived from glucose and fatty acids. They are regarded as green chemicals due to their renewable origin, biodegradability, and low environmental impact. The chemical structure of the AGs contains an amide bond between the hydrophobic and the hydrophilic moieties (Scheme 1), which renders the molecule resistant to the alkaline conditions, a desirable property in surfactants intended for detergent applications. In addition to stability, safety, compatibility and synergism with other surfactants, AGs were used in the formulations of detergent, personal care, and pharmaceutical products [1,2].

Industrial synthesis of fatty acid glucamides involves a two-step reaction: glucose reacts with methylamine in the first step catalysed by Raney nickel to give N-methyl glucamine (MEG), which then reacts with a fatty acid methyl ester to give the fatty acid N-methyl glucamide (amide). Fatty acid glucamides can also be synthesised enzymatically using lipases as catalysts [3,4]. The enzymatic synthesis of AGs using MEG and fatty acid or fatty acid methyl ester yields a mixture of amide (surfactant) and amide-ester as a by-product (see Scheme 1) [5]. The unreacted MEG is an undesirable component in the final product, as it can be a precursor for nitrosamine, a potential carcinogen [6].

A sensitive, simple, and validated analytical method is thus required to monitor the reaction and its components, especially the MEG content. GC analysis is not suitable since MEG has high melting temperature and boiling point (130˚C and 490˚C, respectively). LC-MS-based analytical method has been used for monitoring the AGs in environmental and biodegradation studies [7,8]. A rapid HPLC method with UV detection or refractive index (RI) detection has also been reported [9], however in our experiments, we could not detect MEG using either UV and/or RI detectors.

In the present paper, we describe the development of an HPLC method to monitor the enzymatic synthesis of N-lauroyl-N-methyl glucamide, and we show that ELSD was a superior alternative to UV and RI detection. The method has been validated and the safety, health and environmental impacts of the chromatographic method were assessed using the HPLC-EAT tool, the environmental-assessment software developed earlier by us for evaluating the greenness of the HPLC methods [10].

## Results and discussion

### Reaction and detector selection

In order to develop an enzymatic solvent-free reaction for the synthesis of N-lauroyl-N-methyl glucamide, lauric acid and/or methyl laurate were used as acyl donors or the hydrophobic component of the surfactant, and MEG was used as the acyl acceptor or the hydrophilic part. The use of lauric acid and methyl laurate alone with MEG in 1:1 molar ratio did not provide good reaction conditions; the former produced a highly viscous homogenous salt-complex while the latter led to the precipitation of MEG at the start of the reaction. Hence, lauric acid was required to keep MEG in solution, and methyl laurate lowered the viscosity and avoided the use of organic solvents in the reaction medium while also serving as acyl donor. Optimization of the reaction was based on variation of the molar ratio of lauric acid and/or methyl laurate to MEG [4]; the latter was always set to be the limiting substrate in the reaction (see the section: Monitoring the synthesis of N-lauroyl-N-methyl glucamide).

To monitor the formation of the amide in this reaction using a HPLC method, the challenge is to separate compounds that are differing in polarity, i.e. MEG is highly polar, lauric acid and amide-ester are highly nonpolar while amide has intermediate polarity.

**Figure 1:**
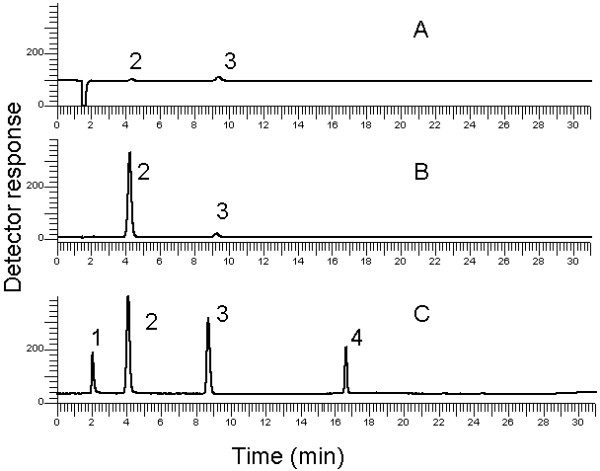
**Comparison of HPLC analyses of the reaction components in a crude reaction mixture from the synthesis of N-lauroyl-N-methyl-glucamide by isocratic method using UV (A) and RI (B) detectors, and the developed gradient method using Evaporative Light Scattering Detector ELSD (C).** Solutes: MEG: 1; amide: 2; lauric acid: 3; amide-ester: 4. Isocratic method: mobile phase: methanol: water: TFA 75:25:0.3 v/v ; flow rate 1 ml min^−1^ and UV wavelength set at 210 nm.

A reaction mixture sample containing the substrates, amide and amide-ester, was chromatographed on a reverse phase column and analysed using three different detectors: UV, IR, and ELSD. Isocratic elution program (methanol: water: TFA, 75:25:0.03 v/v) was adopted with UV and RI detectors, while a gradient elution (Table 1) was applied in case of ELSD. A comparison of chromatograms obtained using the three detectors is shown in Figure 1. Two of the four analytes were detected using the UV detector, as seen in Figure 1A. The absorbance of saturated fatty acid or fatty acid methyl esters arises mainly from the carbonyl group, which accounts for the poor response in UV detection. MEG did not absorb and amide-ester was not eluted during the run time and consequently not detected.

**Table 1:** Chromatographic conditions adopted for gradient HPLC separation of reaction components in the enzymatic synthesis of N-lauroyl N-methyl glucamide

**Mobile phase**	Solvent A =	Water: trifluoroacetic acid (0.05% w/w)	
	Solvent B =	Methanol	
**Gradient**	Time (min)	% Solvent A	% Solvent B
	0	25	75
	5	25	75
	10	5	95
	10	5	95
	5	25	75
	10	25	75
**Flow rate**	1 ml/min		
**Column**	LiChrospher® 100 RP-18 (5 μm) (LiChroCART® 125-4 HPLC cartridge) Merck, Darmstadt, Germany		
**Column temperature**	40°C		
**Detection**	ELSD (Alltech 3300, Alltech Associates, USA)		
**Injection volume**	5 ul		
**ELSD settings**			
**Temperature**	38°C		
**Gas flow (air)**	1.3 L/min		
**Gain**	1		
**Run time**	40 min		

RI is a universal detector, but its main limitation is that it cannot be combined with gradient elution. Isocratic elution certainly prolongs the analysis time, especially for compounds that are strongly adsorbed to the column. RI detector could only detect two of the four analytes present in the mixture: the amide and the fatty acid (Figure 1B). Notably, the detector response was very weak for the fatty acid, and it was not possible to detect MEG and amide-ester peaks. MEG has probably the same refractive index as the mobile phase. Since the amide-ester with two fatty acid moieties was very strongly adsorbed to the column, it could not be detected (Figure 1B). ELSD allowed the identification of the four analytes (Figure 1C), enabling a shorter run time and faster analysis. This is due to the possibility of gradient elution, and the absence of interference of the solvent front peak.

### Optimisation of the ELSD settings

**Figure 2:**
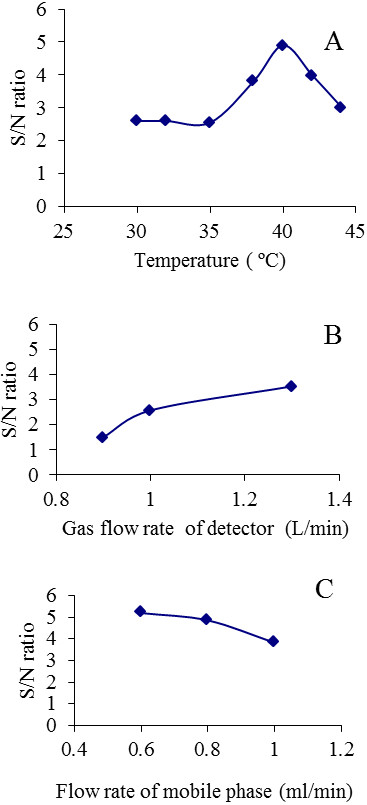
**ELSD optimisation for HPLC analysis of solutes.** Methyl laureate was used as analyte for the optimization experiment. The Signal over Noise ratio (S/N) was monitored regarding the following factors: the detector temperature **(A)**, the gas flow rate in the detector **(B)**, and the flow rate of the mobile phase **(C)**. Standard conditions are mobile phase flow rate of 1 ml/min, 1.3 L/min gas flow to the detector, detector temperature is 38°C, and gain of the detector is set to 1.

The signal intensity of analyte peaks observed by ELSD detector is highly dependent on different factors including the chromatographic conditions such as the flow rate, mobile phase composition, and settings of the detector itself. To optimize these conditions, methyl laurate was chosen as the analyte for optimization of the ELSD settings as it is the most volatile and most difficult to detect among all the analytes. Three parameters were varied: detector temperature, gas flow rate (nebulizer), and mobile phase flow rate (See Figure 2), and their effect on the Signal/Noise (S/N) ratio was monitored. S/N ratio has to be higher than 3 or 5 for detection or quantification purposes. As seen in Figure 2, temperature of the detector had a substantial effect on S/N ratio. The detector temperature directly affected the volatilization of the carrier mobile phase solvent. The temperature settings of 38 and 40°C showed good S/N ratio for methyl laurate detection (Figure 2A). The effect of pumping the filtered air into the detector at three flow rates was tested on the S/N ratio, and 1.3 L/min was chosen as the optimum for the detection (Figure 2B). The flow rate of the mobile phase was observed to have a significant effect on the S/N, low flow rates allowed good S/N ratio at the expense of the elution time (Figure 2C). The final mobile phase flow rate chosen was 1 ml/min as it gave a good compromise between S/N ratio and the overall chromatographic run time.

### Optimisation of the chromatographic conditions

To obtain optimum separation between the peaks of MEG, lauric acid, amide and amide-ester, different parameters, i.e., mobile phase composition, pH, and length of the chromatographic column, were studied. Various mobile phases with different methanol concentrations (90%, 80%, 75% and 70% v/v) were tested. Mobile phase containing 75% methanol and LiChrospher® 100 RP-18, 150 mm column were finally chosen. The amide product could be easily separated from the ester formed as a minor product (Scheme 1) inspite of similarity in molecular weight, structure and adsorption properties. However, amide-ester was better eluted using 95% v/v methanol. Hence, using a methanol gradient allows the separation of all the components. A satisfactory chromatographic profile for analysis of the reaction mixture of amide surfactant synthesis was obtained with ELSD detector (Figure 1C). In the design of the gradient mode, we chose a mobile phase composition of 75% methanol in the first 5 minutes of the run to allow sufficient peak resolution of the amide and the ester peaks, and subsequently a gradient with increasing methanol content up to 95% was set over 10 minutes followed by holding for another ten minutes (Table 1). During the gradient run, peak 4 (amide-ester) eluted at retention time of *ca.* 17.00 minutes.

### Calibration curves

The response of the ELSD detector is known to be nonlinear. Logarithmic (base 10) transformation of the analyte amount and the ELSD response was described to obtain a linear relationship [11,12]. Polynomial curve fitting of the ELSD detector responses was also reported; quadratic and third degree equations were described for the curve fitting with ELSD detectors [13].

Table 2 shows the calibration equations for the four analytes based on correlation between the logarithm of peak areas and the logarithm of concentrations and the corresponding R^2^ values. In case of MEG the second order polynomial equation (not shown in Table 2) and log-log plotting showed good R^2^ values 0.999 and 0.9959, respectively. The limits of detection and quantification were based on the ratio of Signal/Noise. S/N = 3 and S/N = 10 respectively, were calculated for MEG, amide and amide-ester (see Table 2). MEG could be detected starting from 0.12 μg, which reflects the sensitivity of the method to monitor this critical compound in the final product. The detection limit of the amide (0.1 μg equivalent to 20 μg/ml) was slightly higher than the previously reported values obtained with related non-ionic surfactants [14].

**Table 2:** Calibration of the ELSD response of the four analytes based on the relationship of logarithmic values of both peak areas and analyte concentrations

**Peak no.**	**Compound**	**Rt**	**Equation**	**R**^**2**^	**LOD (μg)**	**LOQ (μg)**	**Linear range**	**Precision (R.S.D.%, n = 5)**	
								**Intra-day**	**Inter-day**
1	MEG	2.18 ± 0.14	y = 1.4507x + 2.3077	0.9959	0.12	0.49	0.49-6.20	4.01	3.25
2	Amide	4.31 ± 0.30	y = 1.3716x + 2.3961	0.9986	0.10	0.59	0.59-5.90	2.54	2.24
3	Lauric	9.15 ± 0.48	y = 2.1981x - 1.5648	0.9846	2.02	5.25	5.25-26.23	3.62	2.98
4	Amide ester	17.04 ± 0.35	y = 1.5903x + 1.4929	0.9987	0.04	0.11	0.11-4.52	2.07	1.95

The precision of the analytical method was determined using intra- and inter-day variability measurements. Solutions of a defined concentration of reference compounds were tested. For intra-day variability, the samples were examined in triplicates three times within 1 day, while for inter-day variability, the samples were analysed in triplicates for consecutive 3 days. The obtained relative standard deviations were less than 5% (Table 2).

### LC-MS identification of the unknown compounds

**Figure 3:**
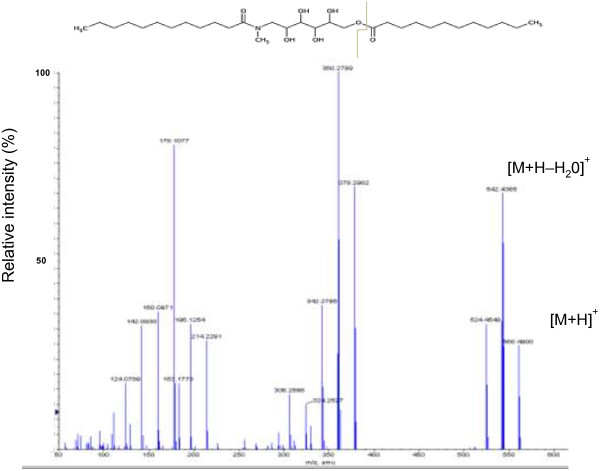
Positive mode LC-MS-MS chromatogram for the confirmation of amide-ester (Mw = 559).

Liquid Chromatography Electrospray Ionisation (LC ESI) was used to confirm the detected HPLC peaks 3 and 4 (Figure 1C). The mass spectra showed formation of sodium adduct ion in positive ion detection (Table 3). The amide and amide-ester molecules do not contain acidic or basic functional groups, and thus association with other ions in the solution is expected under electrospray ionization conditions. In the positive mode ionization mode, the amide and amide-ester compounds showed an abundant [M + H]^+^ ion accompanied by small intensity of loss of water peak [M-H_2_0]^+^ which was also noticed as a major fragment in the collision induced dissociation step. Figure 3 shows the LC-MS-MS applied in positive ionization mode for the amide-ester. The obtained fragmentation patterns were highly consistent with their chemical structures (Table 3). These findings are in agreement with the results of Gonzalez *et al*. and Eichhorn *et al*. [7,8].

**Table 3:** Major ions observed by positive ESI-MS of amide and amide-ester peaks and the major fragments detected by LC-MS-MS of these two compounds

**Compound**	**m/z**	**Major fragments**
**Amide (M = C**_**19**_**H**_**39**_**NO**_**6**_**)**	377	
[M + H]^+^	378	360, 196, 78
[M + Na]^+^	400	382
[M + H–H_2_0]^+^	360	178, 164
**Amide-ester (M = C**_**31**_**H**_**61**_**NO**_**7**_**)**	559	
[M + H]^+^	560	360, 542, 178
[M + Na]^+^	582	
[M + H–H_2_0]^+^	542	360
[2 M + Na]^+^	1141	582

### Environmental assessment using HPLC-EAT software

The developed method was evaluated further with respect to environmental impact using HPLC-EAT (Environmental Assessment Tool), an easy-to-use software providing an indication about the environmental, health and safety impacts of the chromatographic method [10]. HPLC-EAT calculates the impact of the solvents used in the analytical method and presents an output score reflecting the greenness of the method. The higher the score the less green is the method. The tool has been successfully used for evaluation of different analytical as well as preparative chromatographic methods. An advantage of HPLC-EAT over the other software package EATOS, is that it has a built-in data on risk parameters of organic solvents to do the assessments and allows reproducible assessment results [10].

**Figure 4:**
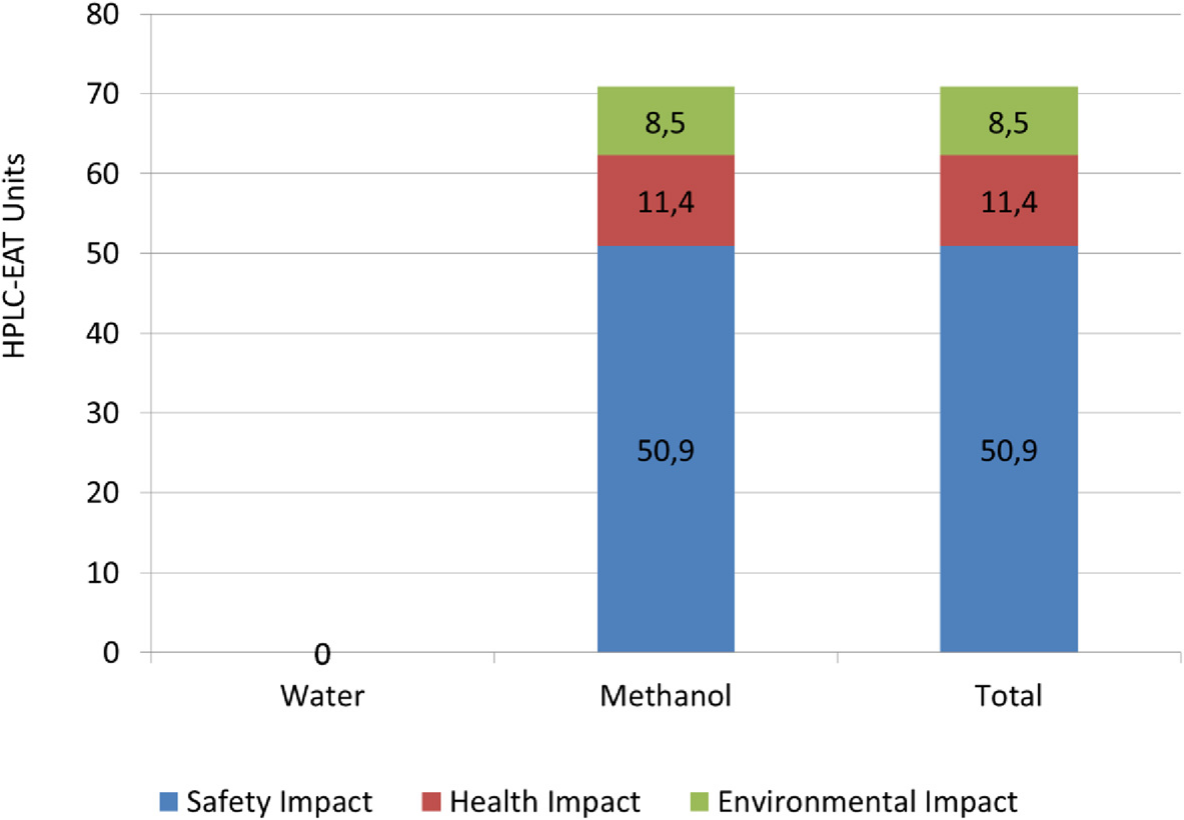
**Evaluation of environmental, health and safety impact of the chromatographic method described in Table **1** using HPLC-EAT software.**

For the HPLC method developed in this report, HPLC-EAT gave a score of 73 HPLC-EAT units. Methanol represented 36 ml of the 40 ml mobile phase consumed during the chromatographic run, and this solvent amount contributed for the safety, health and environmental impact shown in Figure 4. The bar chart shows that the safety impact of the method is a major contribution to the overall impact that is related to methanol being a flammable solvent. HPLC-EAT does not assign penalties to use of water. In our previous study, HPLC-EAT of different analytical methods developed to analyse different types of surfactants showed a score ranging from to 43 to 182 [10]. Based on this comparison, we can state that our method is environmentally acceptable and lies within the HPLC-EAT score range of HPLC methods described for surfactants analysis.

### Monitoring the synthesis of N-lauroyl-N methyl glucamide

The HPLC method described here was successfully used to quantify the enzymatic synthesis of N-lauroyl-N methyl glucamide. The method allowed good separation and quantification of main reaction analytes i.e., the limiting substrate (MEG), the product (amide) and the by-product (amide-ester). Different molar ratios of MEG, lauric acid and/or methyl laurate were mixed and the reactions were catalysed by an immobilized lipase preparation. When lauric acid and methyl laurate were used together as substrates, the methyl laurate content in the reaction was rapidly decreased after the start of the reaction (Figure 1C). This is due to the lipase mediated hydrolysis of methyl laurate to lauric acid even under the solvent free conditions and is noted as the disappearance of methyl laurate peak in Figure 1C. A solvent-free reaction including lauric acid, methyl laurate and MEG in a 1:1:1 molar ratio resulted in N-lauroyl-N methyl glucamide yield of 34% with amide-ester as a major by-product. The final reaction mixture was subjected to hydrolysis under mild alkaline conditions to give a final amide yield of 99% and the excess acylating agent remained as laurate that can be recycled for subsequent reactions. The HPLC method allowed the monitoring of the reaction time course since MEG is the limiting substrate of the reaction. It could also confirm the full conversion of MEG since it showed high sensitivity (LOD = 0.12 μg).

## Materials and methods

### Reagents and chemicals

Novozym®435 (immobilised lipase from *Candida antarctica* of 10,000 Propyl Laurate Units (PLU) per gram), was a gift from Novozymes (Bagsvaerd, Denmark). N-methyl-glucamine (MEG) was purchased from Sigma. HPLC-grade methanol, lauric acid and trifluoroacetic acid for spectroscopy were purchased from Merck. Methyl laurate was procured from Fluka. Milli-Q (Millipore, Milford, MA, USA) quality water was used. N-Lauroyl-N-methyl glucamide (amide) and O-lauroyl-N-lauroyl methyl glucamide (amide-ester) were produced in-house enzymatically and purified using flash chromatography according to Maugard et al. [15]. Structure confirmation was done using infrared and mass spectroscopy.

### HPLC apparatus and chromatographic conditions

HPLC from PerkinElmer Series 200 system equipped with a binary pump, autosampler, oven, interface (NCI 900), and three detectors: UV from PerkinElmer 785A, RI from Hitachi L-2490 and ELSD from Alltech (3300, Alltech Associates, USA), was used. ELSD was operated in a temperature range of 25°C to 45°C and a gas flow of 1.3 L/min and gain of 1. N-Methyl glucamide, fatty acid and their products were separated on an LiChrospher® 100 RP-18 HPLC column, with a guard cartridge RP-18 from Merck, Darmstadt, Germany. Aqueous solution of methanol was used as mobile phase and the injection volume was 5 μl.

### Enzymatic synthesis of N-methyl-N-lauroyl glucamine

N-Methyl glucamine (3.5 mmol) was mixed with lauric acid (3.5 mmol) and methyl laurate (3.5 mmol total) in a round bottom flask, and the reaction was run in solvent-free medium under stirring at 90°C. Novozym®435 at 4% w/w of total substrates weight was added as catalyst in all reactions. The details of the reaction were reported elsewhere [4].

### Mass spectrometry

Mass spectrometry of O-lauroyl-N-lauroyl methyl glucamide (amide-ester) was conducted on a hybrid QS-STAR Pulsar quadrupole TOF mass spectrometer (PE Sciex Instruments, Toronto, Canada). The spectrometer was connected with a similar LC-HPLC system. The electrospray ionization (ESI) source was set to positive ion mode. The quadrupole system was adjusted to scan between m/z 100–2000 in TOF-MS mode whereas for product ion mode (i.e., MS/MS) a range of m/z 50–2000 was chosen. Data was assessed using the Analyst® QS software (PE Sciex Instruments, Toronto, Canada).

## Conclusion

Monitoring the enzymatic synthesis of the surfactant N-lauroyl-N-methylglucamide was achieved by a HPLC method with ELSD. This method is a better alternative to the previously reported HPLC method using UV or RI detectors. It was very sensitive for detecting MEG (LOD = 0.12 μg), which enables the detection of trace amounts of the compound in the final surfactant product. Calibration curves of the different analytes using ELSD as detector were made using double-logarithmic (log-log) relation. The greenness profile of the method was evaluated using HPLC-EAT software and was found to be acceptable. The method was successfully used to monitor solvent-free synthesis of the surfactant, which is free from the substrate MEG.

## Abbreviations

AGs: Alkyl glucamides; MEG: N-methyl glucamine; Amide: N-lauroyl-N-methyl glucamide; TFA: Trifluoroacetic acid; UV: Ultra-violet; RI: Refractive index; ELSD: Evaporative light scattering detector; LOD: Limit of detection; LOQ: Limit of quantification; TOF: Time of flight; S/N: Signal over noise ratio; HPLC-EAT: HPLC-environmental assessment tool.

## Competing interests

The authors declare that they have no competing interests.

## Authors’ contributions

YG did the experiments and wrote the manuscript, COÅ, supervised the experiments and suggested use of ELSD and discussed the results, RHK supervised the whole project and revised the manuscript. All the authors read and approved the final manuscript.
